# Cross-Camera External Validation for Artificial Intelligence Software in Diagnosis of Diabetic Retinopathy

**DOI:** 10.1155/2022/5779276

**Published:** 2022-03-09

**Authors:** Meng-Ju Tsai, Yi-Ting Hsieh, Chin-Han Tsai, Mingke Chen, An-Tsz Hsieh, Chung-Wen Tsai, Min-Ling Chen

**Affiliations:** ^1^Department of Ophthalmology, Taoyuan General Hospital, Ministry of Health and Welfare, Taoyuan, Taiwan; ^2^Department of Ophthalmology, National Taiwan University Hospital, Taipei, Taiwan; ^3^Acer Medical Inc., New Taipei, Taiwan; ^4^Hsieh's Endocrinologic Clinic, New Taipei, Taiwan; ^5^Department of Internal Medicine, School of Medicine, National Defense Medical Center, Taipei, Taiwan; ^6^Joy Clinic, Taoyuan, Taiwan; ^7^Chen Min Ling Medical Clinic, New Taipei, Taiwan

## Abstract

**Aims:**

To investigate the applicability of deep learning image assessment software VeriSee DR to different color fundus cameras for the screening of diabetic retinopathy (DR).

**Methods:**

Color fundus images of diabetes patients taken with three different nonmydriatic fundus cameras, including 477 Topcon TRC-NW400, 459 Topcon TRC-NW8 series, and 471 Kowa nonmyd 8 series that were judged as “gradable” by one ophthalmologist were enrolled for validation. VeriSee DR was then used for the diagnosis of referable DR according to the International Clinical Diabetic Retinopathy Disease Severity Scale. Gradability, sensitivity, and specificity were calculated for each camera model.

**Results:**

All images (100%) from the three camera models were gradable for VeriSee DR. The sensitivity for diagnosing referable DR in the TRC-NW400, TRC-NW8, and non-myd 8 series was 89.3%, 94.6%, and 95.7%, respectively, while the specificity was 94.2%, 90.4%, and 89.3%, respectively. Neither the sensitivity nor the specificity differed significantly between these camera models and the original camera model used for VeriSee DR development (*p* = 0.40, *p* = 0.065, respectively).

**Conclusions:**

VeriSee DR was applicable to a variety of color fundus cameras with 100% agreement with ophthalmologists in terms of gradability and good sensitivity and specificity for the diagnosis of referable DR.

## 1. Introduction

Diabetic retinopathy (DR) is one of the most severe sight-threatening diseases worldwide. Among patients with diabetes, the prevalence was estimated to be 34.6% for any degree of DR, 7.0% for proliferative DR, and 10.2% for vision-threatening DR [1]. The population of patients with diabetes has been increasing in recent years; however, DR awareness and regular evaluation for DR at recommended time points among these individuals remain suboptimal, probably due to poor compliance and limited resources in some areas [2, 3]. Therefore, the development of a cost-effective screening program for DR using fundus photography is an important issue for both patients and healthcare professionals. In recent decades, various computer programs have been developed for automated analysis of color fundus images with acceptable and comparable accuracy to those of human graders [4, 5]. The sensitivity for the detection of referable DR ranged from 85.0% to 96.8% in these studies. Despite the increased efficiency for DR screening, the software used in the automated analysis largely learns explicit disease features taught by specialists, such as the shape and number of dot hemorrhages shown on the photos, to determine DR severity. The application of specified rules to machine learning may limit the detection of undefined features that exist in retinal images.

In recent years, deep learning algorithms have been developed to work through convolutional neural networks (CNNs) and constantly adjust the internal parameters to optimize the predictive capabilities for image analysis and classification with supervised or unsupervised learning. Deep learning algorithms have been proven to be effective in detecting DR using color fundus photographs with good accuracy [6–10]. However, the results of their application in real-world settings may be less satisfactory [11, 12], partially due to image discrepancies among different races or the use of different fundus cameras [13].

The VeriSee DR (Acer Inc., Taiwan) is a certified image assessment software in Taiwan and Thailand, and it uses CNN as the principle of deep learning algorithms for DR screening [14]. During the development of the deep learning models, local image datasets from Taiwan, which contained single-field, 45-degree color fundus photography taken with a nonmydriatic fundus camera (CR-2 series, Canon Inc., Japan) from Taiwanese diabetes patients (mostly East Asians in ethnicity), were incorporated in addition to the open-access dataset EyePACS for model training. The sensitivity (89.2%) and specificity (90.1%) of VeriSee DR in detecting referable DR during the validation stage of model development were good. The accuracy of this software was also good (sensitivity of 95.0% and specificity of 89.9%) for external validation during the subsequent clinical trial in Taiwan. To extend the applicability of the software, this study is aimed at validating the accuracy of VeriSee DR for its application to fundus images taken with different fundus cameras.

## 2. Methods

### 2.1. Image Datasets

Color fundus images of diabetes patients taken with nonmydriatic fundus cameras, including the TRC-NW series (TRC-NW400, TRC-NW8, TRC-NW8F, and TRC-NW8F plus, Topcon Inc., Japan) and the nonmyd 8 series (nonmyd 8 and nonmyd 8 s, Kowa Inc., Japan) in three general practice clinics were collected for studies. All images were taken without pupil dilatation, and all participants were Taiwanese (East Asians in ethnicity). The details of the camera specification profiles are presented in Table 1. All images met the criteria for VeriSee DR: single-field, 45- or 50-degree color fundus photographs, JPEG or DICOM as image formats, and a resolution of at least 1024 × 1024 (1 M) pixels. Such criteria met the standard for DR diagnosis using color fundus photographs. TRC-NW, TRC-NW8, TRC-NW8F, and TRC-NW8F plus used the same camera module and had the same image output formats, except for the additional function of fluorescein angiography in TRC-NW8F and TRC-NW8F plus. Similarly, the nonmyd 8 and nonmyd 8 s had the same camera module and image formats, except for the additional function of anto-fluorescence photography in Kowa nonmyd 8 s. Therefore, the camera models were classified into three categories (TRC-NW400 series, TRC-NW8 series, and nonmyd 8 series) for validation.

After image data collection, only images containing both the disc and central fovea were included. One ophthalmologist then performed the screening process to determine if the image quality was good enough for the diagnosis of DR, and images that were judged as ungradable were excluded. Finally, a total of 1407 fundus photographs were enrolled: 477 of the TRC-NW400 series, 459 of the TRC-NW8 series, and 471 of the nonmyd 8 series. This study adhered to the tenets of the Declaration of Helsinki. It was approved by the National Taiwan University Institutional Review Board (No: 201706108RIPC) with waiver of informed consent.

### 2.2. Grading for Diabetic Retinopathy

All fundus photographs were graded by three board-certified ophthalmologists based on the International Clinical Diabetic Retinopathy Disease Severity Scale [15]. Referable DR was defined as moderate nonproliferative DR (NPDR) or worse, and the images were judged as either “referable” or “non-referable” by each ophthalmologist. The final diagnosis was based on majority voting from three ophthalmologists, which served as the gold standard for this study.

### 2.3. Validation

VeriSee DR was applied to all fundus images for the diagnosis of referable DR. First, the VeriSee DR would judge if the image was gradable. VeriSee DR would further determine if the image was either “referable DR” or “non-referable” if the image was gradable; otherwise, the image would be tagged “ungradable.” The diagnostic results from VeriSee DR were then compared with those of the gold standard.

### 2.4. Statistical Analysis

The sensitivity and specificity for the diagnosis made by VeriSee DR were calculated for each image series, and simple asymptotic formulas based on the normal approximation to the binomial distribution were used to estimate the 95% confidence intervals. The results from the three camera models and the results of the original camera model (Canon CR-2 series) were compared using the Chi-square test. The results would be considered as “qualified” if a sensitivity of more than 87% and a specificity of more than 85% were achieved.

## 3. Results

After enrolment, 1407 fundus photographs were collected, including 477 from the TRC-NW400 series, 459 from the TRC-NW8 series, and 471 from the nonmyd 8 series. All enrolled images (100%) were judged as “gradable” by VeriSee DR and were sent for further diagnosis of referable DR. Among the 1407 fundus photographs, 239 (17.0%) were diagnosed by ophthalmologists as “referable.”

### 3.1. Diagnostic Accuracy among Different Camera Models

According to a previous study, the sensitivity and specificity of the original camera model (CR-2 series) were 95.0% and 89.9%, respectively. As for the results of the three camera models validated in this study, the sensitivity ranged from 89.4% to 95.7%, and the specificity ranged from 89.3% to 94.2% (Table 2). There was no significant difference in sensitivity (*p* = 0.40) or specificity (*p* = 0.065) among the four camera models. All camera models were judged as “qualified” for the use of VeriSee DR in DR diagnosis.

## 4. Discussion

For image-assessment deep learning algorithms, capturing images using different cameras is a primary cause of misidentification [16]. FDA-approved AI-based image-assessment algorithms are usually restricted to images taken with certain machines. Therefore, cross-camera external validation is needed if image assessment software is applied to images of different camera models. In the present study, we demonstrated that the VeriSee DR could be applied to fundus photography taken with other camera models for DR screening with relatively good sensitivity and specificity and is comparable to the original camera model. The gradable rate was 100% for images taken from all camera types.

During the model development stage, a supervised training method was used for the model training of VeriSee DR. Instead of learning the explicit features of DR that have been defined, the deep learning algorithms only received information of the DR staging for each color fundus image. Another feature of the VeriSee DR is that it was pretrained with open-access datasets EYEPACS, which contained images taken from various races using various fundus cameras, and then fine-tuned using the image datasets in Taiwan, which contained images mainly from Taiwanese using the same fundus camera model. Such model training methods not only improve the accuracy but also extend the applicability to various clinical situations and camera models.

It is worth mentioning that all enrolled fundus photographs were judged as “gradable” in the present study, meaning that the VeriSee DR was able to grade all the images that were deemed gradable by the clinicians. During the model training, the algorithms of VeriSee DR also received images recognized as “ungradable” by the ophthalmologists, so it could learn to distinguish if the images were gradable or ungradable, similar to the standard of ophthalmologists. This highlights an important point that this deep learning software acquires the ability to screen images that are not perfect enough, for example, with poor contrast or artifacts. Such images are often encountered in daily practice, but they can often be graded correctly by experienced ophthalmologists. The ability of VeriSee DR to simulate human graders can increase the cost-effectiveness of DR screening. We believe that deep learning algorithms using datasets with mostly high-quality images for model training may not be truly representative of real-world conditions and could possibly result in the overestimation of screening performance [17].

The design for VeriSee DR was aimed at the detection of referable DR. Eyes with mild NPDR only will be diagnosed as nonreferable. Our previous study demonstrated that when using the original camera model (Canon CR-2 series), VeriSee DR also had good sensitivity and specificity in detecting any DR which included mild NPDR [14]. In this study, we found that the sensitivity for any DR was good for all three camera models, but the specificity was poorer, which mainly came from the overdiagnosis of mild NPDR (Table 3). One possibility is that trivial artifacts from different cameras might be misinterpreted as small microaneurysms. However, such misinterpretation was limited so that it did not interfere with the diagnosis of referable DR, in which more extensive lesions should exist.

**Figure 1:**
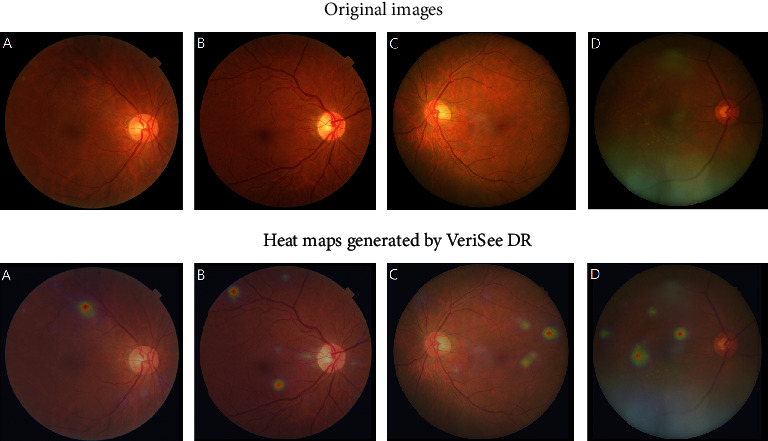
Original images and the heat maps generated by VeriSee DR of 4 cases of proliferative diabetic retinopathy (PDR) or severe nonproliferative diabetic retinopathy (NPDR) that were diagnosed as nonreferable DR by VeriSee DR. (a) A case of psedumed PDR with only suspected laser scars but no microaneurysm or hemorrhage, (b) a case of presumed PDR with atypical neovascularization of the disc and few microaneurysms, (c) a case of branch retinal vein occlusion which was diagnosed as severe NPDR, and (d) a case of age-related macular degeneration and old retinal vein occlusion which was diagnosed as severe NPDR.

Although the sensitivity for cross-machine external validation was high in this study, there were still 2 images of PDR and 2 severe NPDR were diagnosed as nonreferable DR by VeriSee DR. We think that these 4 cases were worth further investigation. Among these 2 images of PDR, 1 was diagnosed as PDR due to the presence of some laser scars, but no other DR-related lesions were noted. The other one was diagnosed as PDR due to neovascularization of the disc; however, the neovascularization of the disc was not typical for PDR, and only few microaneurysms were noted, which was well recognized by VeriSee DR. For the 2 cases of severe NPDR, one had dot hemorrhage, hard exudate, and intraretinal microvascular abnormality located only at superior temporal quadrant, which were well recognized by VeriSee DR; after reevaluation, the graders rediagnosed this case as BRVO with collateral vessels. The other one was diagnosed as severe NPDR due to the presence of intraretinal microvascular abnormality and hard exudate; after reevaluation, however, the graders rediagnosed it as age-related macular degeneration and old RVO since they found only collateral vessels, drusen, and RPE changes but no microaneurysm or hemorrhage in this case. The original images and heat maps generated by VeriSee DR of these four cases were shown in Figure 1. Finally, although the results of cross-machine external validation revealed high accuracy in diagnosing referable DR for all three different camera machines, it does not mean that VeriSee DR is applicable for all other camera machines that have not been validated. Further evaluation is needed before we apply VeriSee DR in the images taken from other camera machines.

In conclusion, the VeriSee DR automated screening system is applicable to a variety of color fundus cameras (TRC-NW400, TRC-NW8 series, Kowa nonmyd 8, and Kowa nonmyd 8 s) with 100% agreement among the ophthalmologists in gradability and relatively high sensitivity and specificity for the diagnosis of referable DR.

## Figures and Tables

**Table 1:** Profiles of the color fundus cameras for validation with VeriSee DR.

Brand	Model	Image format	Resolution (pixels)	Angle (degrees)
Topcon	TRC-NW400	DICOM	5 M	45
	TRC-NW8	JPEG, TIFF, PNG, BMP	20 M	45
	TRC-NW8F			
	TRC-NW8F plus			
Kowa	Nonmyd 8	JPEG	24 M	45
	Nonmyd 8 s			

**Table 2:** Sensitivity and specificity of VeriSee DR in diagnosing referable DR in three different camera models.

Fundus camera model	Sensitivity (95% CI)	Specificity (95% CI)
TRC-NW400	89.4% (80.6-98.2%)	94.2% (92.0-96.4%)
TRC-NW8 series	94.7% (89.6-99.8%)	90.4% (87.4-93.3%)
Nomyd 8 series	95.7% (92.1-99.4%)	89.3% (86.0-92.5%)

CI: confidence interval.

**Table 3:** Sensitivity and specificity of VeriSee DR in diagnosing any DR in three different camera models.

Fundus camera model	Sensitivity (95% CI)	Specificity (95% CI)
TRC-NW400	92.7% (87.5-97.9%)	61.7% (56.8-66.6%)
TRC-NW8 series	92.9% (88.4-97.4%)	73.9% (69.2-78.6%)
Nomyd 8 series	95.1% (92.0-98.2%)	56.9% (51.2-62.7%)

## Data Availability

Dataset will be available under request.

## References

[1] Yau J. W., Rogers S. L., Kawasaki R. (2012). Global prevalence and major risk factors of diabetic retinopathy. *Diabetes Care*.

[2] Sabanayagam C., Yip W., Ting D. S., Tan G., Wong T. Y. (2016). Ten emerging trends in the epidemiology of diabetic retinopathy. *Ophthalmic Epidemiology*.

[3] Ting D. S., Cheung G. C., Wong T. Y. (2016). Diabetic retinopathy: global prevalence, major risk factors, screening practices and public health challenges: a review. *Clinical & Experimental Ophthalmology*.

[4] Tufail A., Rudisill C., Egan C. (2017). Automated diabetic retinopathy image assessment software: diagnostic accuracy and cost-effectiveness compared with human graders. *Ophthalmology*.

[5] Abràmoff M. D., Folk J. C., Han D. P. (2013). Automated analysis of retinal images for detection of referable diabetic retinopathy. *JAMA Ophthalmology*.

[6] Ting D. S. W., Pasquale L. R., Peng L. (2019). Artificial intelligence and deep learning in ophthalmology. *The British Journal of Ophthalmology.*.

[7] Abramoff M. D., Lou Y., Erginay A. (2016). Improved automated detection of diabetic retinopathy on a publicly available dataset through integration of deep learning. *Investigative Ophthalmology & Visual Science*.

[8] Gulshan V., Peng L., Coram M. (2016). Development and validation of a deep learning algorithm for detection of diabetic retinopathy in retinal fundus photographs. *JAMA*.

[9] Gargeya R., Leng T. (2017). Automated identification of diabetic retinopathy using deep learning. *Ophthalmology*.

[10] Ting D. S. W., Cheung C. Y., Lim G. (2017). Development and validation of a deep learning system for diabetic retinopathy and related eye diseases using retinal images from multiethnic populations with diabetes. *JAMA*.

[11] van der Heijden A. A., Abramoff M. D., Verbraak F., van Hecke M. V., Liem A., Nijpels G. (2018). Validation of automated screening for referable diabetic retinopathy with the IDx-DR device in the Hoorn diabetes care system. *Acta Ophthalmologica*.

[12] Verbraak F. D., Abramoff M. D., Bausch G. C. F. (2019). Diagnostic accuracy of a device for the automated detection of diabetic retinopathy in a primary care setting. *Diabetes Care*.

[13] Ting D. S. W., Peng L., Varadarajan A. V. (2019). Deep learning in ophthalmology: the technical and clinical considerations. *Progress in Retinal and Eye Research*.

[14] Hsieh Y. T., Chuang L. M., Jiang Y. D. (2021). Application of deep learning image assessment software VeriSee™ for diabetic retinopathy screening. *Journal of the Formosan Medical Association*.

[15] Wilkinson C. P., Ferris F. L., Klein R. E. (2003). Proposed international clinical diabetic retinopathy and diabetic macular edema disease severity scales. *Ophthalmology*.

[16] Zhong Z., Zheng L., Zheng Z., Li S., Yang Y. (2017). Camera style adaptation for person re-identification. http://arxiv.org/abs/1711.10295.

[17] Nielsen K. B., Lautrup M. L., Andersen J. K. H., Savarimuthu T. R., Grauslund J. (2019). Deep learning-based algorithms in screening of diabetic retinopathy: a systematic review of diagnostic performance. *Ophthalmology Retina*.

